# Building up tomorrow

**DOI:** 10.1016/S1808-8694(15)31276-3

**Published:** 2015-10-20

**Authors:** Henrique Olival Costa

Some days ago I read on the editorial of *Folha de Sao Paulo* newspaper what may represent a collective expectation of our poor emerging society.

Talking about the topic for the writing assignment at FUVEST (college admission test for public universities in Sao Paulo), the journalist wrote: ‘FUVEST has really gone too far this time. It has asked applicants in this year test to prepare an essay about “The program for *unratcheting* life“, yes, you got it right. Let me explain: last year a group of activists named “Contra File”, placed an old ratchet wheel over a pedestal at Largo do Arouche, downtown Sao Paulo. The installation was named “Monument to the invisible ratchet wheel”. It was an attempt to criticize, according to the authors, “the biopolitical control that is exerted through visible and/or invisible forces” that we are all subject to. Based on that, FUVEST asked applicants to make a statement about “excessive control of all varied types that are exerted over people's bodies and minds”. What did examiners intend? That applicants would discourse about the one-dimensional man described by Marcuse? Or address the microphysics of power described by Foucault? Or advocate the “wild good”? Frankly… I feel like being somewhat rude: ask someone who lives in Guaianazes (outskirts of Sao Paulo) what he thinks about the *unratcheting* of society while he is on a crowded bus. In an attempt to awaken the critical sense of applicants, the writing assignment induced only to divagation and unsubstantiated clichés.’

I can not refrain myself from saying that I was taken aback by the negative approach of the journalist in view of the attempt to find an alternative metaphorical meaning to an invented word based on a real fact. It seems that Guimaraes Rosa is no longer a reference to create, add up and deconstruct words, generating new meaning and colors to human activity, which became an alienating agent of society. However, how does it relate with our own specialty? Maybe it has a lot to do with it if we analyze our own educational structure, considering medical school, specialization or even graduate studies. We were based on quite a fruitful and at the same type safe model of promoting education, in which the Socratic form of tutorship was expanded and modified, maintaining direct transmission of knowledge and permanent discussion of reports and experiences; however, we introduced a high number of participants in those groups, not allowing them to participate in one-to-one discussions.

We live today in a highly hierarchical system in which applicants go to college already ranked by a second-year sophomore and take every step of his/her development in undergraduate school under the influence of informal tutors, reaching residence programs under a head of the service and learning how to be ordered about and obey, many times without even speaking up against unacceptable things. Unfortunately, it shows in our scientific meetings, in which few have questions or comments during or after the presentations, because discussions are limited to panelists, whose opinions are never openly contested. The same submissive model is repeated in scientific opinions and reviews of the Brazilian Journal of Otorhinolaryngology and the responses of authors. Everything is lifeless, shallow, colorless and non-criticized…

Once Gonzaguinha (popular Brazilian composer) wrote “I'll fight for the youth that does not wait but builds our dreamed tomorrow”.

At a time in which we are rethinking the format and content of medical residence programs in our specialty, I believe we have a lot to consider about the way we want to prepare physicians to professional life. For a long time, physicians were the people sought for advice, a role model, known to be reliable by the society. It used to be so because it could be like that. No one was prohibited to think, give opinions, discuss, debate, participate in important decisions, and above all create expectations by renewing status quo based on past experiences, defined by metaphorical meditations and social concerns that were not criticized but rather expected and looked up by peers and clients.

Where have those physicians gone? Currently we have to fight for our medical fees, compete for clients and referrals, advocate our right to practice medicine without interference from related areas. But isn't it a way of making us become fair, responsible and respected? Aren't we being forced to leave behind our superior and sovereign position over our activities because we did not maintain our humanist perspective, personal formation and own opinions?

There is a lot to give though to - we have already compromised significantly but we are not mere healthcare technicians. Or are we?

